# Cross-Sectional Association between the Number of Missing Teeth and Cardiovascular Disease among Adults Aged 50 or Older: BRFSS 2010

**DOI:** 10.1155/2014/421567

**Published:** 2014-01-30

**Authors:** R. Constance Wiener, Usha Sambamoorthi

**Affiliations:** ^1^Department of Dental Practice and Rural Health, School of Dentistry, and Department of Epidemiology, School of Public Health, Robert C. Byrd Health Sciences Center, West Virginia University, P.O. Box 9448, Morgantown, WV 26506, USA; ^2^Department of Pharmaceutical Systems and Policy, Robert C. Byrd Health Sciences Center, West Virginia University, School of Pharmacy, P.O. Box 9510, Morgantown, WV 26506-9510, USA

## Abstract

*Objective*. The relationship between oral health and cardiovascular disease is an emerging area of research. The objective of the current study is to evaluate the association of cardiovascular disease and the number of missing teeth as a risk indicator. *Methods*. Cross-sectional study design with data on 275,424 respondents aged 50 or older from the 2010 Behavioral Risk Factor Surveillance System survey was used. The dependent variable was self-reported cardiovascular disease. The association between the number of missing teeth and cardiovascular disease was analyzed with multivariable logistic regression. The regression was adjusted for sex, race/ethnicity, age, education, income, dental visits, smoking status, physical activity, and body mass index. *Results*. In our study sample, 9.9% reported edentulism. Cardiovascular prevalence rates for those with edentulism were 25.4% and for those without any missing teeth were 7.5%. Respondents who reported edentulism teeth were more likely to report cardiovascular disease (AOR = 1.85, 95% CI = 1.71, 2.01). *Conclusion*. There was an independent association between the number of missing teeth and cardiovascular disease even after controlling for a comprehensive set of risk factors. These findings highlight the need to explore the potential role the number of missing teeth have in the risk of cardiovascular disease among older adults.

## 1. Introduction

The lifetime incident risk for cardiovascular disease is very high. It is estimated at 66% for men and 50% for women at the age of 40. Numerous studies have reported that healthy lifestyle practices (maintaining a normal blood pressure, having a normal body mass index, limiting alcohol use, and smoking cessation or never smoking) and good psychological health (such as stress management, and social support) can reduce the risk of cardiovascular disease [1–5]. Additionally, poor oral health has been recognized as a potential risk factor to developing cardiovascular disease due to its systemic impacts. However, a recent review conducted by the American Heart Association concluded that, while there is an association between periodontal disease and atherosclerotic vascular disease, there may not be a causative relationship. The review also identified knowledge gaps in the understanding of oral health and atherosclerotic vascular disease [6]. The knowledge gaps included limited understanding of the relationship of the number of missing teeth and cardiovascular disease.

Understanding the association between the number of missing teeth and the risk of cardiovascular disease is important because oral health interventions may improve cardiovascular health. Biological mechanisms proposed for an association of the number of missing teeth and cardiovascular disease include (1) inflammation, (2) infection, and (3) diet and nutrition [7]. For the inflammation hypothesis, chronic oral infection (as periodontal diseases leading to the number of missing teeth) contributes to systemic inflammation and increases in the plasma concentration of acute-phase proteins (such as C-reactive protein), inflammatory cytokines, (such as interleukin-6), and coagulation factors (such as fibrinogen) which increase the potential for cardiovascular disease [8]. The long, chronic exposure is thought to result in cardiovascular changes that persist after tooth extraction. In support of the oral infection hypothesis, it is known that, oral bacteria, bacterial components, or bacterial end products enter the blood stream and result in transient bacteremias [8]. Additionally, oral bacterial DNA has been detected in endarterectomy samples by polymerase chain reactions (PRC) [8]. The third hypothesis, diet and nutrition, is based on the dysfunctional masticatory system and on the ability to obtain proper nutrition from the diet [7, 9]. Researchers conducting a study of 749 participants over the age of 40 in Korea indicated an association between posterior missing teeth and an increase in intima-media thickness of the common carotid artery [10]. A similar study of 131 participants indicated that the number of missing teeth and maximal aortic intima-media thickness was significantly correlated [11].

Indeed, a handful of studies from the United States (US) and other countries indicated a relationship between the number of missing teeth and increased cardiovascular disease. A study in the US of 41,407 adult professional men (using the Health Professionals Follow-Up Study) and 58,974 adult female nurses (using the Nurse Health Study) reported that the participants with 0–10 teeth had a relative risk of coronary heart disease of 1.36 for men and 1.64 for women compared with the participants who had 25 or more teeth [12]. Research using the Glasgow Alumni Cohort indicated that, compared to men with 25–32 teeth at baseline, men with 0–10 teeth had a significantly higher risk of coronary heart disease suggesting that there was an increased risk of CVD for participants with 9 or more lost teeth (adjusted hazard ratio, 1.35) [13]. A study surveying participants of the Northern Finland Birth Cohort from 1997 to 1998 showed that an increased number of missing teeth was not associated with the presence of angina pectoris (adjusted odds ratio: 1.53, 95% CI: 0.69, 3.42) [14]. A study set in Brazil involving 382 consecutive patients undergoing coronary angiography showed an association between poor self-reported oral health, the number of missing teeth, and atherosclerosis [15]. The adjusted prevalence ratio was 1.22 (95% CI: 1.02, 1.19) [15].

However, to our knowledge, there has not been a nation-wide study examining the association between the number of missing teeth and cardiovascular disease among older adults in the US. Such a study with large numbers and a diverse population would help alleviate concerns of spurious findings existing in previous studies [8]. Therefore, the primary objective of the current study was to examine the relationship between the number of missing teeth and cardiovascular disease prevalence in adults aged 50 years and above in the United States. The research hypothesis is that the number of missing teeth will be associated with greater risk of cardiovascular disease in older adults compared to older adults who do not have any missing teeth.

## 2. Materials and Methods

### 2.1. Study Design

This study is a secondary data analysis of public access data which does not require IRB approval. It used a retrospective, observational, and cross-sectional design using data from the annual 2010 Behavioral Risk Factor Surveillance System (BRFSS) survey of Americans.

### 2.2. Data Source

The researchers at the Centers for Disease Control and Prevention (CDC) in conjunction with the state health departments actively survey the population who are 18 years or older in a nationally representative study, the BRFSS survey, for health behaviors and health risk factors. The survey-developers from the CDC and state health department created probability samples of households with a multistage cluster design to provide a nationally representative sample with a complex design. The interviews are conducted over the telephone using random-digit dialing in blocks of telephone numbers to create the probability sample of households. The sampling design is detailed elsewhere [16]. There were 451,075 participants in the 2010 BRFSS survey. The eligible participants had no missing data on the presence or absence of cardiovascular disease, had no missing data on the number of missing teeth that they had, were 50 years and older, and had completed the interview. There were 6,541 participants who had missing data on the number of missing teeth that they had. The final sample size was 275,424.

### 2.3. Dependent Variable

The dependent variable was the presence of cardiovascular disease (yes/no). This was determined by an affirmative response to either of the posed questions in the BRFSS survey: “Has a doctor, nurse, or other health professional ever told you that you had a heart attack, also called a myocardial infarction?” “Has a doctor, nurse, or other health professional ever told you that you had angina or coronary heart disease?”

### 2.4. Key Independent Variable: The Number of Missing Teeth

The number of missing teeth was derived from self-report. The posed question and statements were “How many of your permanent teeth have been removed because of tooth decay or gum disease? Include teeth lost to infection, but do not include teeth lost for other reasons, such as injury or orthodontics. If wisdom teeth are removed because of tooth decay or gum disease, they should be included in the count for lost teeth.” The possible responses were “none,” “1 to 5,” “6 or more, but not all,” and “all.” We maintained the categories presented in the BRFSS. The threshold of five missing teeth has been shown to reduce misclassification of missing teeth extracted for orthodontics or the third molar extractions [9].

### 2.5. Other Independent Variables

Other independent variables or factors were demographic characteristics—sex (women, men); race/ethnicity (non-Hispanic Blacks and others, Hispanic, and non-Hispanic White); age (50–59 years, 60–69 years, and 70 years and above); socioeconomic characteristics—education (less than high school, some college or technical school, high school, graduate of college or technical school, or above); annual household income from all sources (less than $15,000, $15,000–$24,999, $25,000–$34,999, $35,000–$49,999, $50,000, and above); use of oral health care (dental visit within the year (yes, no)); life style practices—smoking status (never smoker, current smoker, and former smoker); physical activity (“During the past month, other than your regular job, did you participate in any physical activities or exercises such as running, calisthenics, golf, gardening, or walking for exercise?” (yes, no)); body mass index (BMI) categories (normal—less than 18–25 kg/m^2^, overweight—25–30 kg/m^2^, and obese—>30 kg/m^2^).

Analyses were conducted using SAS 9.3 (Cary, NC) software using BRFSS survey weights provided to adjust for unequal selection probabilities, over sampling, and nonresponse. Bivariate, chi square tests were applied to determine if there were significant relationships between the exposure of interest (the number of missing teeth categories) and the other variables. Multivariable logistic regression was used to evaluate the association between the number of missing teeth and cardiovascular diseases after controlling for other risk factors described above.

## 3. Results

The sample included 275,424 participants. The majority were female (53.8%), non-Hispanic white (77.5%), and between the ages of 50 and 59 (42.9%). Most had some college, technical school, or above (61.4%). Data are not reported in tabular form. Income above $50,000 was reported by 46.1%, and income below $15,000 was reported by 10.6%. There were 71.5% of the participants who reported having had a dental visit within the year; 50% reported never smoking; 72.1% reported moderate physical activity; 31.6% reported normal BMI.

The unweighted numbers and weighted percentages of the number of missing teeth categories by subject characteristics are presented in Table 1. In our study sample 9.9% reported that all of their teeth were missing; 18.4% reported that greater than 6 missing teeth were missing but not all of their teeth were missing; 36% reported 1 to 5 missing teeth; 35.2% reported no missing teeth. Statistically significant associations (*P* < 0.0001) were observed between categories of the number of missing teeth and sex, race/ethnicity, age, education, income, having had a dental visit within the previous year, smoking, physical activity, and body mass index. Statistically significant differences in proportion with cardiovascular disease were observed for all risk factors. In terms of cardiovascular disease prevalence, among those who reported no missing teeth, only 7.5% reported having cardiovascular disease compared to 25.4% among those with edentulism. Similarly, among those who had greater than or equal to 6 missing teeth but not all teeth missing, the prevalence of cardiovascular disease was higher (19.2%) compared to those without any missing teeth (7.5%). A significantly higher proportion of those with 1–5 missing teeth reported having cardiovascular disease (11.8%) compared to those without any missing teeth.

Adjusted odds ratios (AOR) and 95% confidence intervals (CI) from multivariable logistic regression of the number of missing teeth categories, sex, race/ethnicity, age, education, income, having had a dental visit within the previous year, smoking, physical activity, and BMI categories on the likelihood of cardiovascular disease are displayed in Table 2. For adults who lost 1 to 5 teeth compared to adults who had no missing teeth, the AOR for cardiovascular disease was 1.27 (95% CI, 1.19, 1.35). The AOR for cardiovascular disease for the participants with 6 or more missing teeth, but not all missing teeth was 1.65 (95% CI, 1.54, 1.77), and the AOR for cardiovascular disease for the participants who had all of their teeth missing was 1.85 (95% CI, 1.71, 2.01). We also observed that adults who visited the dentist were less likely to report cardiovascular disease compared to those who did not visit dentists in the past year. The AOR was 0.88; 95% CI = 0.83, 0.93.

In our study, other risk factors were also associated with increased risk of cardiovascular disease. Adults who were women, old age, overweight, obese, and being a current smoker were more likely to report cardiovascular disease compared with adults who were men, belonged to younger age-group, had normal BMI, and reported never smoking. Adults who reported regular physical activity and had a greater income had a reduced likelihood of cardiovascular disease compared to adults without regular physical activity and lower income groups.

## 4. Discussion

This study indicated a significant independent relationship between edentulism and cardiovascular disease in a large population of older adults aged 50 years or older in the US as compared with individuals who had no missing teeth. We also observed increased likelihood of cardiovascular disease among those with some missing teeth. Older adults with 1 to 5 missing teeth and greater than 6 missing teeth, but not all teeth missing, were more likely to report presence of cardiovascular disease as compared with older adults who had no missing teeth. Findings from the current study are consistent with those conducted in Northern Finland, Brazil, and Scotland and other studies conducted in the US [12–15].

The increased risk of cardiovascular disease and the number of missing teeth can be attributed to many factors including nutritional problems. The number of missing teeth has been associated with chewing difficulty [7, 9], and chewing difficulty has been associated with dietary changes, self-reported nutritional problems, and decreasing the range of food eaten [17]. Heart-healthful diets have been researched and have been found to lower blood pressure in controlled feeding studies, for example, the Dietary Approaches to Stop Hypertension (DASH) and the three Optimal Macronutrient Intake Trial to Prevent Heart Disease (OmniHeart) diets (higher carbohydrate, higher protein, higher unsaturated fat) [18]. In a randomized controlled study of two modified Mediterranean diets and a low fat control diet (in which participants did not have controlled feeding), results indicated that a Mediterranean diet supplemented with extra-virgin olive oil or nuts was associated with better survival, which was similar to those results of the Women's Health Initiative Dietary Modification Trial [4]. These diets and others stress the importance of a variety of foods, particularly fruits and vegetables, and the avoidance of highly processed foods, particularly refined carbohydrates as being strongly supported in cardiovascular health maintenance [19]. Eating fruits and vegetables is problematic for many people, particularly people who have many missing teeth. The number of missing teeth may therefore increase the risk for several systemic diseases, including cardiovascular disease [20].

In addition to diet and nutrition, inflammation and infection have been proposed for the association between the number of missing teeth and cardiovascular disease [7]. Previous research has indicated that chronic infection, as seen in chronic periodontal diseases, has systemic consequences in increasing inflammatory cytokines and coagulation factors that are associated with cardiovascular disease which are thought to remain increased even after tooth extraction [8]. Another potential mechanism involves oral bacteria (or their products) entering the vascular system to create transient bacteremias which potentiate cardiovascular disease [8].

An interesting finding of our study is the reduced risk of cardiovascular disease among those who had visited the dentist compared to those who had not visited dentists in the past year. Additional study is needed to determine if periodontal care reduces systemic inflammation and its potential cardiovascular effects. Although studies have documented the importance of preventive oral health among those with teeth, our study findings highlight the importance of the number of missing teeth as an important risk factor in the health care of older adults. Improved oral health is critical; however, many older adults find financial barriers (no dental insurance and inability to pay) as access to care barriers [21].

This study has a number of strong points. It uses recent nationally representative data from a large, national, US data source. A comprehensive list of risk factors was included in the logistic regression model to address residual confounding. However, our study findings need to be interpreted in the light of its limitations. As a characteristic of all cross-sectional study designs, although associations can be determined, temporal associations and causality cannot be verified. This study is also based on self-reported data, with the potential of misclassification.

Despite the limitations, our study extends previous epidemiological research by highlighting the association between edentulism and cardiovascular disease [9]. The current study adds to the growing but nascent literature on the association between the number of missing teeth and cardiovascular disease. Additionally, the study findings have clinical implications. The significant association between the number of missing teeth and cardiovascular disease in an older population suggests (1) the importance of maintaining healthy teeth and gingival tissue into old age and (2) the potential usefulness of physician oral screening for the number of missing teeth and vigilance for cardiovascular disease in the presence of many lost teeth.

## Figures and Tables

**Table 1:** Characteristics of the 275,424 study participants according to the number of missing teeth in the behavioral risk factor surveillance system, 2010 (*N*, weighted%).

	No missing teeth		1–5 missing teeth		6 or more, but not all missing teeth		All teeth missing		Chi-square *P* value
	*N*	wt%	*N*	wt%	*N*	wt%	*N*	wt%	
Participants	90023	35.2	97745	36.5	53718	18.4	33938	9.9	*P* < 0.0001

Cardiovascular disease	7063	2.6	11828	4.3	10415	3.5	8484	2.5	*P* < 0.0001
No CV disease	82960	32.6	85917	32.2	43303	14.8	25454	7.4	

Female	57151	19.2	59852	18.9	33411	10.0	22212	5.7	*P* < 0.0001
Male	32872	16.0	37893	17.6	20307	8.4	11726	4.2	

NHW	78872	29.6	80194	27.5	41231	13.0	26916	7.4	*P* < 0.0001
NHB	2801	1.4	6681	3.3	6265	2.6	3256	1.2	
Hispanic	3786	2.7	5494	3.9	2844	1.7	1519	0.7	
Other	3568	1.6	4213	1.8	2640	1.0	1602	0.5	

50–59	40555	19.5	32666	15.9	12544	5.5	5134	2.0	*P* < 0.0001
60–69	28650	9.5	33429	11.3	17770	5.8	10105	2.9	
70 and above	20818	6.3	31650	9.4	23404	7.0	18699	4.9	

Less than HS	3256	1.5	6410	3.0	7948	2.9	9144	2.7	*P* < 0.0001
High school	18957	6.9	30798	10.7	21178	6.9	14550	4.2	
Some college	23365	8.6	27670	10.1	14392	4.8	7114	2.0	
College	44260	18.3	32687	12.8	10085	3.8	3024	1.0	

<$15,000	4491	1.8	8227	3.3	9201	3.2	8061	2.4	*P* < 0.0001
$15K to <$25K	8981	3.3	15069	5.6	12457	4.5	9741	3.2	
$25K to <$35K	7836	2.9	11872	4.4	7428	2.9	4162	1.5	
$35K to <$50K	12131	4.8	14988	6.0	7216	3.0	3207	1.2	
$50K+	44262	22.7	33898	17.3	9896	4.9	2866	1.2	

Dental visit	74667	29.4	76174	28.2	33882	11.6	7042	2.3	*P* < 0.0001
No dental visit	15199	5.9	21411	8.4	19649	6.7	26185	7.5	

Smoker	7046	2.8	11504	4.6	10440	3.8	8225	2.6	*P* < 0.0001
Past smoker	28202	11.0	35200	13.3	22432	7.8	14490	4.2	
Never smoker	54127	21.4	50472	18.7	20526	6.8	11019	3.1	

Diabetes	11415	4.4	17373	6.6	13382	4.8	9573	2.8	*P* < 0.0001
No diabetes	78542	30.8	80289	29.9	40285	13.6	24319	7.0	

PA	71328	28.3	71300	26.9	33562	11.6	18410	5.4	*P* < 0.0001
No PA	18606	7.0	26320	9.7	20079	6.7	15461	4.5	

BMI < 25	32816	12.5	29891	10.9	15293	5.1	10711	3.1	*P* < 0.0001
BMI 25 to <30	33171	14.0	36594	14.6	19239	6.9	11785	3.7	
BMI 30+	20434	8.6	27587	11.0	17416	6.4	10172	3.1	

NHW: non-Hispanic White; NHB: non-Hispanic Black; HS: high school; K: 1000; Dental visit: dental visit within the year; PA: physical activity; BMI: body mass index.

**Table 2:** Adjusted odds ratios (AOR) and the 95% confidence intervals according to cardiovascular diseases for the variables in the final logistic regression analysis behavioral risk factor surveillance system, 2010.

	AOR (CI)	*P* value
Missing teeth		
1–5 extracted-teeth versus no missing teeth	1.27 (1.19, 1.35)	<0.0001
>6 extracted-teeth versus no missing teeth	1.65 (1.54, 1.77)	<0.0001
Edentulism versus no missing teeth	1.85 (1.71, 2.01)	<0.0001
Sex		
Female versus male	0.48 (0.46, 0.50)	<0.0001
Race/ethnicity		
Non-Hispanic Black versus non-Hispanic White	0.87 (0.80, 0.95)	0.0018
Hispanic versus non-Hispanic White	0.84 (0.75, 0.94)	0.0025
Other versus non-Hispanic White	1.17 (1.03, 1.32)	0.0132
Age		
60–69 versus 50–59	1.89 (1.78, 2.02)	<0.0001
70 and older versus 50–59	3.32 (3.12, 3.54)	<0.0001
Education		
HS versus less than HS	0.96 (0.89, 1.04)	0.3278
Some college/technical versus less than HS	1.06 (0.97, 1.15)	0.2101
College/technical degree versus less than HS	0.95 (0.87, 1.04)	0.2717
Income		
$15000–25000 versus less than $15000	0.83 (0.77, 0.89)	<0.0001
$25000–35000 versus less than $15000	0.65 (0.60, 0.71)	<0.0001
$35000–50000 versus less than $15000	0.61 (0.56, 0.67)	<0.0001
Greater than $50000 versus less than $15000	0.49 (0.45, 0.53)	<0.0001
Dental visit within the year		
Yes versus no visit	0.88 (0.83, 0.93)	<0.0001
Smoking		
Current smoker versus never smoker	1.46 (1.36, 1.57)	<0.0001
Former smoker versus never smoker	1.42 (1.35, 1.49)	<0.0001
Physical activity		
Yes versus no	0.75 (0.71, 0.79)	<0.0001
Body Mass Index		
Overweight versus normal	1.25 (1.18, 1.32)	<0.0001
Obese versus normal	1.66 (1.57, 1.76)	<0.0001
